# Stress Septal Sign (Triple S) Preexists in Hypertensive Hearts and Clarifies Critical Diagnostic Strategies

**DOI:** 10.3390/jcm14176143

**Published:** 2025-08-30

**Authors:** Fatih Yalçin, Boran Cagatay, M. Roselle Abraham, Mario J. Garcia

**Affiliations:** 1 Department of Cardiology, UCSF HEALTH, School of Medicine, Cardiac Imaging, San Francisco, CA 94158, USA; boran.cagatay@ucsf.edu (B.C.); roselle.abraham@ucsf.edu (M.R.A.); 2 Cardiology Division, Montefiore Einstein Center for Heart and Vascular Care, Bronx, NY 10461, USA; mariogar@montefiore.org; 3 Department of Medicine, Division of Cardiology, Albert Einstein College of Medicine, Bronx, NY 10461, USA

**Keywords:** hypertension, stress septal sign, Triple S, left ventricular remodeling, basal septal hypertrophy

## Abstract

The interventricular septum is recognized as the first region to undergo remodeling, and a septal bulge is described as an early echocardiographic sign of hypertensive heart disease. Using third-generation microscopic ultrasonography in an animal model, we validated, for the first time, that remodeling originates in the basal septum, presenting as basal septal hypertrophy (BSH), an early imaging biomarker, and subsequently progresses to other regions, leading to tissue dysfunction and heart failure. We have termed this finding the “stress septal sign” (Triple S) because a variety of stress stimuli, such as treadmill exercise or pressure overload from aortic banding in animals, induced BSH, a region with more intensive sympathetic innervation than the mid-apex. This finding also represents a conjunctive point between functional etiologies, such as hypertension, and emotional etiologies that precipitate acute stress cardiomyopathy. Microscopic analysis of the remodeling revealed that hemodynamic stress has a specific effect on cardiac geometry. The Triple S is associated with exercise-induced hypertension and high stress scores in patients with hypertension. Furthermore, three-dimensional segmental remodeling is more effective than cross-sectional measurements for detecting the impact of superimposed multiple stressors. A high-rate pressure product and blood pressure variability in patients exhibiting the Triple S should be managed comprehensively through an integrated approach to stress and hypertension to avoid high mortality in clinical practice. A precise etiologic evaluation of incidentally detected BSH may contribute to the early diagnosis of hypertensive disease. The integrated and timely management of stress and hypertension is important for patients presenting with the Triple S and high stress scores. This management strategy may provide a practical solution for avoiding the adverse hypertensive consequences of global remodeling and maladaptation to superimposed multiple stressors.

## 1. Introduction

There is a general consensus regarding the importance of the septal bulge in hypertension; in fact, it has been pointed out that the septal bulge represents an early echocardiographic sign in hypertensive heart disease [1,2]. A potential mechanism for the development of focal distortion over the septal base could be related to increased wall stress in the basal cavity compared to the mid-apical region, since the left ventricular (LV) basal diameter is the largest LV intracavity segment facing the greatest wall stress [3].

### 1.1. Real-Time Three-Dimensional Imaging

**Figure 1:**
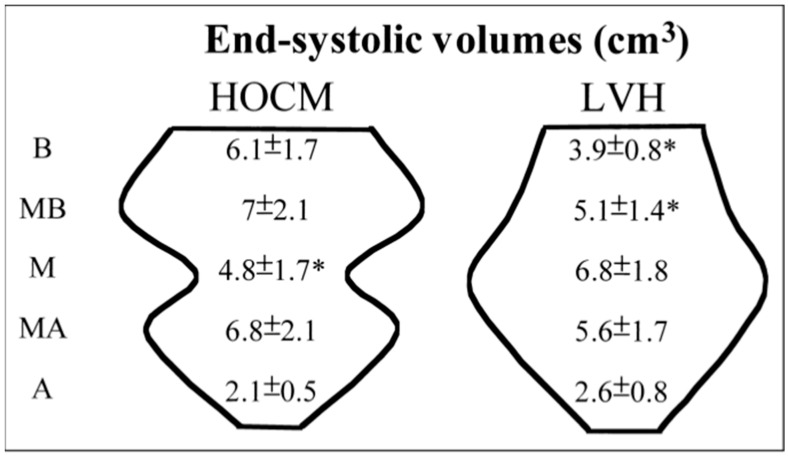
The ampulla shape with a narrowed basal cavity volume analyzed by real-time 3-dimensional segmental cavity volume analyses in LV hypertrophy due to aortic stenosis or hypertension. Reprinted from ref [4]. HOCM: Hypertrophic Obstructive Cardiomyopathy. Values marked with * indicate statistically significant differences.

Segmental LV remodeling analysis by real-time three-dimensional echocardiography (RT3DE) showed non-uniform morphology in patients with pressure overload. We previously developed a quantitative volume index to separate long-axis LV cavities into three equal slices as the base, mid, and apex parts using RT3DE to visualize segmental details of LV cavities and realized that secondary LV hypertrophy is associated with diminished basal intracavity volume like ampulla shape (Figure 1) and preserved LV function, while hypertrophic cardiomyopathy had reduced midsegmental volume representing the catenoid geometry and impaired LV function [4,5].

### 1.2. Stressed Heart Morphology

**Figure 2:**
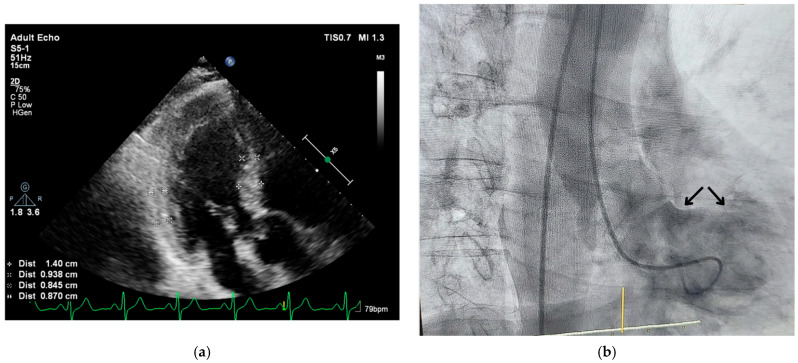
(**a**) A two-dimensional echocardiographic image of a hypertensive patient with a sigmoid septum and ampulla-shaped LV cavity geometry during diastole. Reprinted from ref [9]. (**b**) Preexisting Triple S in the same patient, similar to Takotsubo geometry. Reprinted from ref [9].

We detected basal septal hypertrophy (BSH) in hypertensives and described predominant septal base as the conjunctive point of determination in both acute and chronic conditions, called “stressed heart morphology (SHM)” [6]. In fact, remarkable midapical enlargement with predominant and hyperdynamic LV base in emotionally developed acute stress cardiomyopathy [7] was a consistent finding with LV basal remodeling (Figure 2a,b), as well as hyperdynamic myocardial function at LV base in hypertensive patients [8].

We validated BSH as the early imaging biomarker in both exercise mice and others with transaortic binding [10,11]. This led us to think that exercise may not always be beneficial in patients with SHM [12] and criticized some cardiac nuclear and MRI studies, ignoring blood pressure monitoring in septal remodeling or hyperdynamic LV function [13,14]. Gottdiener et al. previously showed that exercise hypertension could be detected in healthy individuals [15]. We also realized, years later, after validation studies, that our human heterogeneously distributed septal remodeling data were strikingly different from the regular progression of remodeling in animals [16]. Then, we reported the difficulty in determining the certain prevalence of SHM by multimodality imaging [17] and suggested that scientists should be cautious in the quantification of this morphologic finding [18].

### 1.3. Third-Generation Microscopic Imaging

Third-generation microscopic ultrasonographic data in animal validation studies showed that hemodynamic stress independent from etiology is associated with the microscopic remodeling in the early time points prior to LV dysfunction [19]. Beyond the emotional component in acute stress cardiomyopathy and the functional component due to increased afterload in patients with hypertensive heart disease, we have recently mentioned the mechanical component of SHM in aortic stenosis patients [20]. According to these observations, we believe that LV segmental remodeling as detected in hypertensive heart disease could also be clinically important in patients with aortic stenosis [21].

In the recent interesting article reported by Tsuda et al., the authors showed that the analyses of septal morphology leading to a dynamic bulge into the LV outflow tract and dynamic obstruction are extremely important since segmental involvement is the case in a variety of stress stimuli [22]. However, despite 55% of the study population being associated with hypertension and segmental involvement of the septum, for the validated finding as an early imaging biomarker in an animal model with pressure overload stress, the description of cardiac morphology was a normal heart in the conclusion, which seems a limitation of the study [22]. The clinical importance of the early imaging biomarker is primarily related to its potential role in the early treatment of early LV remodeling [16]. Therefore, recent microimaging data are consistent with the clinical reports supporting the paradigm that LV segmental remodeling is possibly accepted as the initiation of hypertensive heart disease [1,2]. In fact, since blood pressure monitoring, especially under pharmacologic stress or during exercise, may be ignored even in trials [12,13,14] and previously undiagnosed hypertension seems not rare even in developed countries [23], the prevalence of hypertensive disease may be underestimated in the population.

### 1.4. Segmental Tissue Function of LV Base

**Figure 3:**
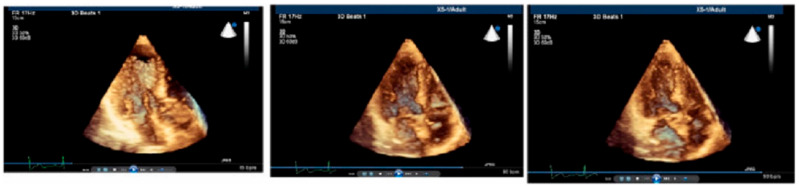
Diastolic BSH and systolic predominately of BSH in a hypertensive by RT3DE. Reprinted from ref [4,19].

**Figure 4:**
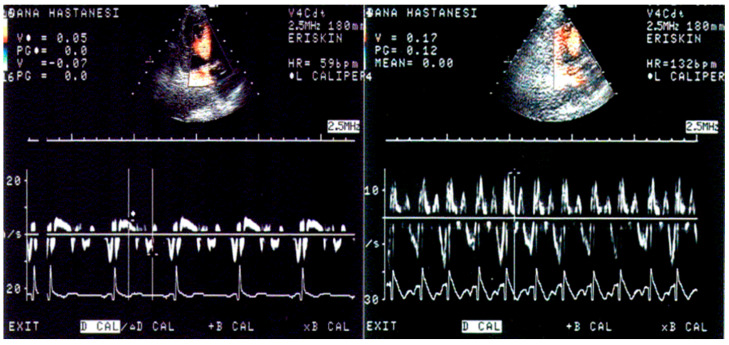
Measurements of maximum basal septal tissue systolic velocities at rest and peak stress in a hypertensive patient with basal septal hypertrophy. Reprinted from ref [4].

Furthermore, we noted in the animal validation studies that the early increment of systolic velocity up to 4 weeks is related primarily to compensatory hyperfunction in the early time points before the development of LV basal dysfunction [10,11]. Similar to humans with hypertensive basal septal hypertrophy (Figure 3) [8], tissue hyperfunction in the animal validation studies was detected by tissue Doppler imaging, which could obviously provide additional benefit to assess the phases of hypertensive disease [17] and quantitative follow-up of hypertensives (Figure 4) in the LV remodeling process [24]. We also suggested that this early abnormality could progress and result in more severe dysfunction of the septal wall compared to the free wall in global LV hypertrophy (LVH) [25].

**Figure 5:**
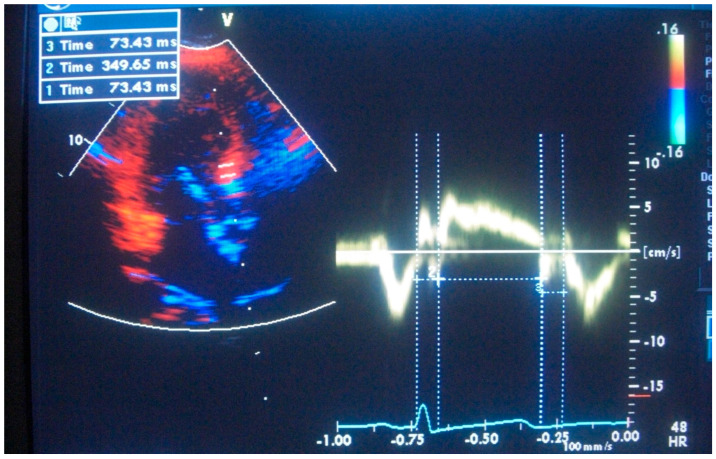
Quantitative segmental LV functional analysis of a hypertensive patient with basal septal hypertrophy using tissue Doppler echocardiography. Reprinted from ref [30].

**Figure 6:**
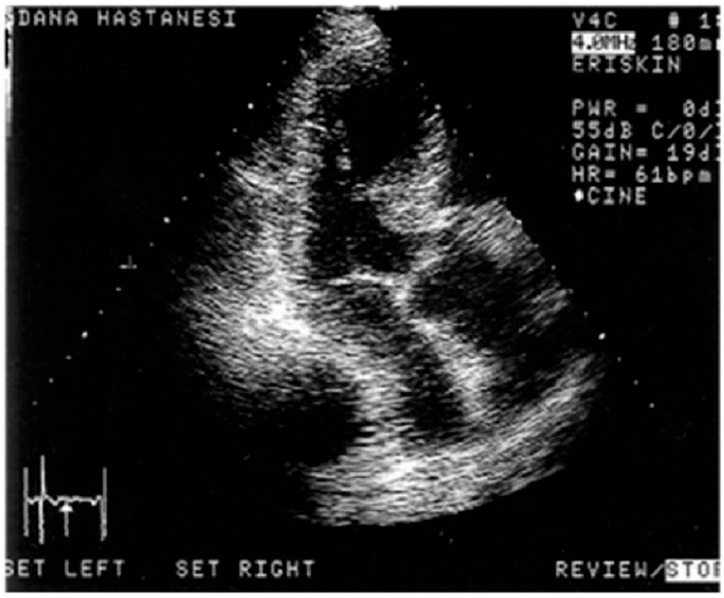
Triple S, which can not be explained by pure hemodynamic overload, is free from activity and related to a high stress score. Reprinted from ref [18].

In fact, septal ablation can be necessary in advanced cases with LV outflow obstruction due to huge septal hypertrophy [26]. It was shown that the LV base has an increased amount of noradrenaline and is associated with a more intensive sympathetic innervation compared to the apical region in animal and human hearts [27,28,29]. Triple S could be a specific location for a variety of stress stimuli because it is an extremely sensitive region to sympathetic overdrive (Figure 5 and Figure 6), which is independent of the type of stress [18,19].

Moreover, regional remodeling before global LVH development might be accepted as an additional stage of LV remodeling in the future, since early biomarkers for hypertension have gained more importance [31,32]. Despite all of the medical efforts on the importance of the early diagnosis of hypertensive heart disease and the general consensus on the clinical importance of BSH, diagnostic success may not be sufficient since 20% of previously undiagnosed hypertensive cases are associated with target organ damage [23].

### 1.5. Triple S and Takotsubo Syndrome

Traditionally classified as an acute and self-limiting cardiomyopathy, Takotsubo syndrome has been widely regarded as a transient response to sudden emotional or physical stress, predominantly in postmenopausal women [33]. However, emerging evidence and refined imaging modalities challenge this purely acute paradigm, suggesting that Takotsubo syndrome may in fact represent the acute manifestation of a chronically evolving myocardial susceptibility [34]. While the clinical presentation is abrupt—often mimicking acute coronary syndrome—the underlying myocardial substrate appears to be shaped over time by a constellation of subtle but significant structural, functional, and hemodynamic alterations [35]. This concept, aligned with the Triple S paradigm, proposes that features such as a small left ventricular cavity, modest basal septal hypertrophy, dynamic intraventricular gradients, and altered ventricular compliance may quietly accumulate, especially in the setting of chronic hypertension or age-related remodeling. These changes may remain clinically silent until an acute stressor—emotional, mechanical, or metabolic—acts as the final trigger, unmasking the vulnerable myocardial phenotype and precipitating the characteristic pattern of apical ballooning. In this light, Takotsubo syndrome is reframed not as a purely reactive process but as a predictable expression of a myocardium rendered fragile by chronic maladaptive remodeling. This model better explains cases occurring in the absence of overt emotional stress, as well as the recurrence of the syndrome in predisposed individuals [36,37]. It also underscores the importance of identifying the chronic substrate—through modalities such as stress echocardiography—rather than focusing solely on acute symptomatology. Recognizing Takotsubo syndrome as the acute endpoint of a long-standing structural and physiological vulnerability has meaningful implications for both diagnosis and long-term management, potentially shifting the clinical focus toward preventative strategies and substrate-directed therapy.

## 2. Why Triple S for Multiple Stressors in Clinical Practice

Triple S—the “stress septal sign” of BSH—often does not correlate in a one-to-one fashion with the severity of pure hemodynamic load. Instead, it appears to arise from the interplay of multiple stress mechanisms. The concept of superposed multiple stressors has been introduced to explain how emotional, mental, physical, and mechanical stress stimuli can converge on the heart to produce focal septal remodeling. In real-world scenarios, acute cardiac events frequently involve combined triggers rather than a single isolated cause.

For example, an episode of extreme emotion may be coupled with sudden exertion, together precipitating a hypertensive surge or stress cardiomyopathy. Accordingly, recent observations emphasize that multiple simultaneous stress inputs (“superposed” stressors) can amplify cardiovascular strain and are likely a major factor in precipitating adverse outcomes.

This paradigm is the rationale behind Triple S: it serves as an early marker of the heart’s maladaptive response to compounded stressors.

In other words, the septal bulge is not merely a marker of long-standing pressure overload but a “conjunctive point of determination” between chronic hypertension (a functional stress) and acute stress cardiomyopathy (an emotional stress).

By recognizing Triple S, clinicians are alerted to an individual’s cumulative stress burden—something that may be missed if one focuses solely on resting blood pressure or a single triggering event.

**Figure 7:**
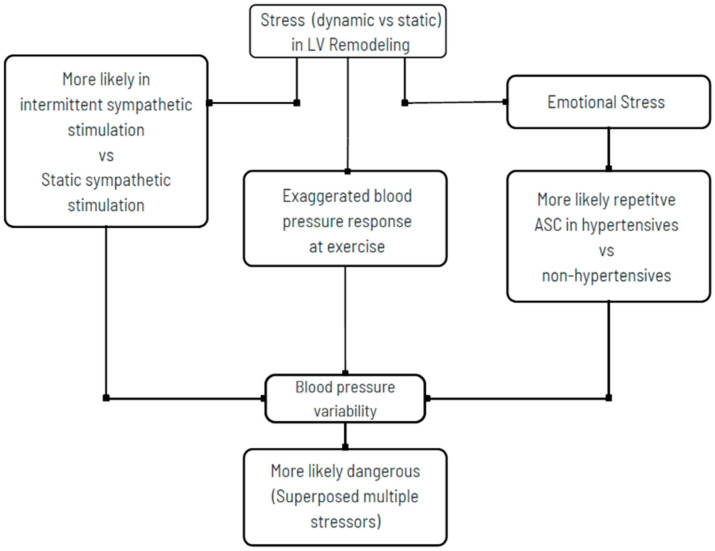
Vascular hazards due to blood pressure variability in a variety of stressors, such as exercise, hypertension, emotional sympathetic overdrive, and a combination of multiple stressors. Reprinted from ref [9].

BSH morphology clinically may not be directly concordant with the severity of hemodynamic overload. Heterogeneity over focal involvement is pretty common in clinical practice and possibly affected by multiple pathogenetic mechanisms [12,17,18,19]. Both long-standing stresses associated with emotional problems and hemodynamic fluctuations under BP variability may not be modulated by parasympathetic balance and restored to the resting values after stress exposure (Figure 7).

We know that early septal remodeling, namely Triple S, can be developed after physiologic exercise in healthy individuals and interpreted to be a physiologically existing phenomenon. However, we clearly detected that blood pressure fluctuations could be neglected even in very precisely conducted National Registries like the LARGE MRI Study, which was supported by the British Heart Foundation [38].

Multiple stressors, at the same time, could be superposed and become clinically dangerous; thus, BSH should be seriously taken into consideration by clinicians as we previously emphasized in the recent books and in scientific exhibitions like the European Society of Hypertension, respectively [39]. Therefore, these reports have given the first messages emphasizing complex SHM developed by superposed multiple stressors, including functional, emotional, or mechanical stress. Both hypertension and anxiety are associated with norepinephrine release and excessive emotional stimulation, even panic attacks. We now know that Triple S is associated with emotional stress in animals, in addition to the pressure overload and physical exercise. These observations may represent a specific location, the septal base, for the nonspecific adaptation of the body to the stress stimuli.

In the validation studies at Johns Hopkins, the morphologic prominence of the LV septal base, which is free tissue from vagal innervation, accounts for half of the total course of global LV remodeling in the small animal model, which supports the underlying chronic hypertension as seen in the predominant septal base in Takotsubo cardiomyopathy. In 2000, we published the ampulla shape using real-time 3D echo at the Cleveland Clinic, which is very similar to LV geometry with that in Takotsubo cardiomyopathy, in which insufficient blood pressure response due to mid-apical microvascular dysfunction, as Messerli described in “Decapitated Hypertension,” may mask the exaggerated hemodynamic response under enormously released neurohormonal blood levels.

As a result, sympathetic overdrive and norepinephrine release are normally associated with hypertension; however, hemodynamic deterioration due to apical ballooning could mask the underlying hypertension clinically in Takotsubo cardiomyopathy. Severity of hypertensive LV hypertrophy is related to norepinephrine release [17], and the predominant involvement of the LV base could possibly be related to the intensive LV basal innervation, which was confirmed histologically [40,41,42]. Consistently, ASC is associated with the predominant LV septal base and excessive emotional stimulation, as well as increased catecholamine levels in the circulation [43].

The role of segmental remodeling involves BSH with dynamic blood pressure fluctuations on exercise [12], which leads to LV cavity dilation [44]. Stress stimuli could be related to a variety of components, including mental, emotional, physical, and mechanical stressors [39]. Superposed stressors take an important role in segmental remodeling, which is the main reason for the existence of “Triple S” in the literature. Incoordination between stress control and hypertension management resulted in an extremely high prevalence of previously undiagnosed hypertension and target organ damage [23,45]. Undiagnosed blood pressure fluctuations due to daily stress lead to high mortality and hemodynamic deterioration in stressful clinical presentations due to emotion-mediated norepinephrine peak levels with acute myocardial decompensation. An interesting part of the clinical picture in Triple S is that both septal bases in hypertensive myocardial tissue and Takotsubo cardiomyopathy give a hyperdynamic response to stress stimuli based on not only visual impression but quantitative analysis of contractility using Doppler tissue imaging [8,46]. Hypercontractile response to sympathetic stimulation is not a rare, common finding in hypertensive patients [8,13,47,48] with LVH, and related hypercontractility may result in diagnostic difficulty in ischemic heart disease [49].

In cross-sectional human data on BSH, the degree of regional hypertrophy over the septal base could be enormous [7,12,17,18,19,39] without any midapical progression compared to regular animal remodeling [10,11]. This discrepancy supports the contribution of the emotional component of stress stimuli and supports the clinical importance of Triple S. In this new paradigm, namely SHM, we have realized that chronic mental, emotional, physical, and mechanical stressors could be superposed on human beings [39] after the determination of heart remodeling findings in animals in microscopic remodeling data. Therefore, years later, we realized that animals that are free of cognitive function have extremely regular segmental remodeling under a variety of stressors, according to Johns Hopkins animal validation studies.

“Scientists themselves don’t always distinguish between the psychological state of stress and the physiological response to stress”, Dr. Ratey JJ. says, adding that chronic stress, which translates emotional stress into physical strain and stress response, can lead to anxiety, depression, as well as high blood pressure [50]. A variety of stressors affect Triple S, the specific location that is free from parasympathetic modulation.

## 3. The Neuro-Cardiac Anatomical and Clinical Importance of Segmental Examination of the Interventricular Septum

The cardiac neural innervation shows asymmetry and heterogeneity. Compelling evidence from anatomical, functional, and imaging studies identifies the basal region of the interventricular septum as a focal point for sympathetic nervous system activity, rendering it particularly susceptible to the effects of stress. The cardiac parasympathetic innervation by the vagus nerve is shown, showing the innervation of both ventricles; it has more influence on the apex compared to the base [51,52]. This condition of less parasympathetic predominance on the basal segment appears to be a key factor in its early and disproportionate involvement in the hypertrophic remodeling process known as SHM.

The anatomical foundation for this sympathetic dominance is well-documented based on studies that have demonstrated that the base of the left ventricle receives greater sympathetic innervation compared to the apex [53]. Histological analysis of the human heart has confirmed a higher density of autonomic nerves in this basal region [42]. This increased innervation is matched by a higher local concentration of catecholamines; specifically, the basal myocardium contains a greater amount of noradrenaline, the primary neurotransmitter of the sympathetic system. This neurochemical arrangement primes the basal septum for a heightened response to sympathetic signals. This has been further substantiated by advanced imaging techniques. Scintigraphy studies utilizing I-meta-iodobenzylguanidine, which is a norepinephrine analog, have shown increased sympathetic activity specifically within the asymmetrically hypertrophied basal septum of patients with hypertension, directly linking the structural change to a state of localized sympathetic hyperactivity [29].

This dense sympathetic innervation has clear and measurable functional consequences. Under conditions of stress, the basal septum exhibits a uniquely hyperdynamic and hypercontractile response. When challenged with a sympathomimetic agent like dobutamine, hypertensive patients with BSH demonstrate a significantly exaggerated response in the hypertrophied segment [48]. Quantitative tissue Doppler imaging shows that maximum systolic velocities in the basal septum at peak stress are significantly higher in patients with BSH compared to individuals with normal cardiac geometry. This hypercontractility is an early sign of the disease process, often preceding global LV dysfunction [8].

The progression from localized sympathetic overactivity to structural change has been prospectively validated in animal models of pressure overload. Using third-generation microscopic ultrasonography, we have confirmed that BSH is the earliest imaging biomarker to appear. This initial remodeling is characterized as a “compensatory hyperactive stage” where the heart adapts to the increased stress [11]. During this phase, elevated intracavitary gradients are observed, reflecting the hyperdynamic function of the early-remodeling heart. However, this adaptive stage is transient. Animal studies show a sharp transition from this state of compensatory hyperfunction to maladaptation, marked by the onset of tissue dysfunction, blunted intracavitary gradients, and the progression to global, concentric LVH [10,17].

The recognition of BSH as the morphological product of a segmental autonomic imbalance has profound clinical implications. It mandates a shift toward the segmental clinical evaluation of the interventricular septum. Global measurements of septal thickness can easily miss or underestimate the significance of BSH, thereby failing to identify patients in the early, adaptive phase of SHM [21]. The incidental discovery of BSH on a routine echocardiogram should not be dismissed as a benign finding. Instead, it should be recognized as a potential marker of underlying, and possibly undiagnosed, hemodynamic or neurologic stress (emotional with high stress score) [39]. Such a finding warrants a more thorough evaluation, including blood pressure monitoring during exercise, to unmask exercise-induced hypertension, which is strongly associated with septal remodeling.

## 4. Conclusions

LV geometric and functional features, with precisely processed etiologic assessment using quantitative cardiovascular imaging, could be widely used in the population and contribute to early medical treatment. Diagnosis of hypertension could be underestimated in early septal remodeling, since it could be missed at rest. Missed hypertension may be related to emotional stress and exercise-induced hypertension. Absence of recorded segmental data globally and hemodynamic overload determination during stress may underestimate the evaluation of heart remodeling and failure in antihypertensive medication. In follow-ups of chronic patients, we strongly believe that this preexisting sign (Triple S) should be recalled, and the optimal beta-blocker dosage should be provided. In fact, this group of medications is effective and used worldwide in the control of myocardial tissue O2 consumption.

## 5. Future Insights

According to the effect of multiple stressors, “stressed heart morphology” may represent an early imaging biomarker in the population with various risk groups [46]. Recently, we have worked on a Special Issue showing imaging and other aspects of the remodeling progression and have summarized the working details of the last two decades in the attached preface of the recent Special Issue (stress septal sign). Animal models using third-generation microscopic ultrasonography have shown that heart remodeling starts on the basal septum. Beyond our description of “microscopic remodeling” in pressure overload and physiologic exercise, animals under emotional stress lead to remodeling of the septum that is free from the vagal nerve. “Superposed multiple stressors”, as the leading killer in the majority of cases, has increased the importance of “segmental remodeling” in cardiac imaging. “Stress septal sign” is the conjunctive point of determination in hypertension as functional and acute stress cardiomyopathy as emotional etiopathologies. Segmental comparative analyses instead of single cross-sections possibly contribute to a neurocardiologic perspective for the combined management of hypertension and stress. These experiences and other published data showed that 60% of chronic diseases are associated with stress. However, only 3% of the population who seek health care find a chance to have specific management for stress. Beyond the clinical aspects of SHM, there is a need to focus on cellular levels of myocardial tissue to investigate whether SHM is the specific location of Selye’s nonspecific general adaptive response to stressors.

However, the industrial design of healthcare is inherently oriented towards costly procedures, potentially resulting in physicians placing excessive emphasis on the device-mediated perspective. In addition, as is the case with advanced phases of diseases, the initiation of treatment, which includes the administration of beta-blockers [54] in conjunction with stress management techniques, could be approached in a more comprehensive manner. This approach is not only cost-effective but also offers a significant public health benefit. Furthermore, there is a strong conviction that SHM could be utilized extensively in the near future from a public health perspective. Combined management of stress and hypertension could control the hazardous effects of superposed multiple stressors, since many cardiac problems can not be explained by classic risk factors; biomarkers could provide additional benefit in clinical practice.

## Data Availability

Not applicable.

## Abbreviations and Acronyms

LV	left ventricle
RT3DE	real-time three-dimensional echocardiography
BSH	basal septal hypertrophy
SHM	stressed heart morphology
Triple S	stress septal sign
LV	LV hypertrophy
